# The generation and evaluation of two panels of epitope-matched mouse IgG1, IgG2a, IgG2b and IgG3 antibodies specific for *Plasmodium falciparum* and *Plasmodium yoelii* merozoite surface protein 1–19 (MSP1_19_)

**DOI:** 10.1016/j.exppara.2012.02.003

**Published:** 2012-04

**Authors:** Jaime R. Adame-Gallegos, Jianguo Shi, Richard S. McIntosh, Richard J. Pleass

**Affiliations:** Lab C4, Institute of Genetics, Queen’s Medical Centre, University of Nottingham, Nottingham NG7 2UH, UK

**Keywords:** Mouse IgG, IgG subclasses, Epitope matched antibodies, Malaria

## Abstract

Murine immunoglobulin G (IgG) plays an important role in mediating protective immune responses to malaria. We still know relatively little about which IgG subclasses protect against this disease in mouse models, although IgG2a and IgG2b are considered to be the most potent and dominate in successful passive transfer experiments in rodent malarias. To explore the mechanism(s) by which the different mouse IgG subclasses may mediate a protective effect, we generated mouse IgG1, IgG2a, IgG2b and IgG3 specific for the C-terminal 19-kDa region of *Plasmodium falciparum* merozoite surface protein 1 (*Pf*MSP1_19_), and to the homologous antigen from *Plasmodium yoelii* (*P. yoelii*), both major targets of protective immune responses. This panel of eight IgGs bound antigen with an affinity comparable to that seen for their epitope-matched parental monoclonal antibodies (mAbs) from which they were derived, although for reasons of yield, we were only able to explore the function of mouse IgG1 recognizing *Pf*MSP1_19_ in detail, both *in vitro* and *in vivo*. Murine IgG1 was as effective as the parental human IgG from which it was derived at inducing NADPH-mediated oxidative bursts and degranulation from neutrophils. Despite showing efficacy in *in vitro* functional assays with neutrophils, the mouse IgG1 failed to protect against parasite challenge *in vivo*. The lack of protection afforded by MSP1_19_-specific IgG1 against parasite challenge in wild type mice suggests that this Ab class does not play a major role in the control of infection with mouse malaria in the *Plasmodium berghei* transgenic model.

## Graphical abstract

**Graphical abstract:**

**Figure:**
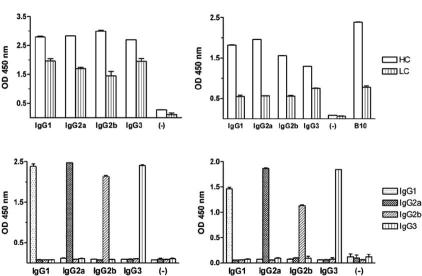

**Highlights:**

► We generated two panels of epitope-matched mouse IgGs anti-*Pf*MSP1_19_ and *Py*MSP1_19_. ► All IgGs were confirmed to be of the correct subclass and bound to the antigens. ► IgG1 triggers *Pf*MSP1_19_-specific NADPH oxidase activation through FcγR cross-linking. ► Passive transfer of IgG1 anti-*Pf*MSP1_19_ did not protect mice from infection.

## 1 Introduction

**Fig. 1:**
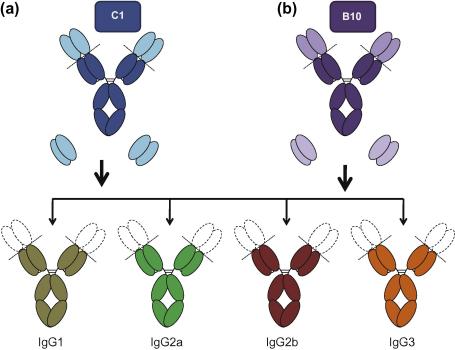
Generating two panels of epitope-matched murine IgG Abs specific for MSP1_19_ epitopes C1 (*P. falciparum*-*Pf*MSP1_19_) or B10 (*P. yoelii*-*Py*MSP1_19_). Diagram represents a general overview to the construction of (a) C1 and (b) B10 mouse IgGs. Variable genes from parental mAbs C1 or B10 were sub-cloned into redesigned expression vectors for each of the mouse IgG subclasses.

Amongst mouse IgG subclasses, IgG2a and IgG2b are considered to be the most potent activators, and dominate in successful passive transfer experiments in both murine infection (including malaria), and murine tumour models (Clynes et al., 2000; Nimmerjahn and Ravetch, 2005; Spencer Valero et al., 1998). Such functional distinctions have been attributed to differences in their capacity to fix complement and/or recruitment of relevant Fc-receptors for IgG (FcγRs) (Nimmerjahn and Ravetch, 2005). However, very few studies have compared epitope-matched IgG subclasses in the context of protection from both communicable and non-communicable diseases (Chu et al., 2010; Giorgini et al., 2008; Ishizaka et al., 1995; McLean et al., 2002; Reitan and Hannestad, 1995, 2002; Rodrigo et al., 2009; Torres et al., 2007), and in these studies, disease-specific effects were observed between IgG subclasses. For example, mouse IgG1 has been shown to be poor at killing tumours, yet plays an important role in controlling gastrointestinal parasites (McCoy et al., 2008; Wojciechowski et al., 2009). Mouse IgG1 is believed not to be a subclass associated with protective properties as it is not a potent complement activator and it possesses an extremely low activation to inhibition (A:I) ratio (0.1 compared to 69 and 7 for IgG2a and IgG2b respectively), as a consequence of its preference for binding the inhibitory FcγRIIB receptor (Woof, 2005). Work with non-epitope matched mouse monoclonal antibodies (mAbs) targeting *Plasmodium yoelii* MSP1_19_ has shown conflicting data, with some IgG1 mAbs protecting from malaria (e.g. mAb G3), and others (e.g. mAb B4), having little or no effect on the course of disease (Spencer Valero et al., 1998). However, in the absence of epitope-matched reagents, it is difficult to directly compare the efficacy of IgG1 against the other subclasses in the protection from blood-stage malaria. Due to structural and functional differences between the murine IgG subclasses (in particular with respect to FcγRs they bind), and to attempt to resolve existing controversies as to whether FcRs are indeed even required for protection from malaria in the mouse, we generated two panels of recombinant mouse IgG1, IgG2a, IgG2b and IgG3 targeting identical epitopes on *Plasmodium falciparum* and *P. yoelii* MSP1_19_ (Fig. 1). These were then used to investigate the anti-malarial properties of the mouse subclasses *in vivo*.

## 2 Materials and methods

### 2.1 Generation of *Pf*MSP1_19_-specific antibodies

#### 2.1.1 Construction of pVL-C1-mouse kappa light chain

For all primer sequences, nucleotides in bold refer to restriction enzyme sites whereas an underline highlights stop codons. The plasmid pVL-C1-mouse kappa was generated by a three step PCR. Primer pairs VL-C1for (5′ GGC ***GTG CAC*** TCC GAT ATT GTG ATG ACC CAG 3′) and k-joint-rev (5′ ggg aag atg gat aca gtt ggt gca gca tca gtt c 3′) were used to amplify the VL by PCR from pVKExpress C1, whilst introducing an *Apa*L1 restriction site on the 5′ end. A second PCR with primer pairs k-joint-for (5′ gaa ctg atg ctg cac caa ctg tat cca tct tcc c 3′) and MK-rev (5′ g***ac tag t***cc ctc taa cac tca ttc ctg ttg a 3′) containing a *Spe*I were used to amplify the mouse kappa light chain constant region from BALB/c genomic DNA. Overlap PCR with primer pairs VL-C1for and MK-rev were then used to fuse as a single PCR product the VL of C1 and the mouse kappa constant region, which was then cloned in pCR2.1-Topo (parking vector) prior to sub-cloning into the parental pVKExpress C1 (McIntosh et al., 2007; Persic et al., 1997) as an *ApaL*I/*Spe*I fragment.

#### 2.1.2 Construction of pVH-C1-mIgG1

pVH-C1-IgA1 (Shi et al., 2011) was digested with *Sal*I to remove the IgA constant region sequence and re-ligated to produce pVH-C1-delIgA1. This plasmid was then digested with *BssH*II/*Xba*I to release the VH of C1 which was then sub-cloned into pcDNA3.1-C1-hIgG1 from which the C1 and hIgG1 sequences were previously deleted (pcDNA3.1-delC1delIgG1) using the same restriction sites to create pcDNA3.1-C1-delhIgG1. The murine IgG1 constant region was amplified from BALB/c genomic DNA using primer pairs Mg1-for (containing an *Xba*I site) (5′ GCA AAA GAG CGG CCT ***TCT AGA*** AGG TTT G 3′) and Mg1-rev (5′ CAC TGG GAT CAT TTA CCA GGA GAG TG 3′), and cloned into the parking vector, prior to sub-cloning as an *Xba*I fragment into pcDNA3.1-C1-delhIgG1 to generate pVH-C1-mIgG1.

#### 2.1.3 Construction of pVH-C1-mIgG2a

Primer pairs Mg2a-for (5′ ***G GTC ACC*** ATC AAG AGG AGG AAG 3′) and Mg2a-rev (5′ ***TCT AGA*** GCT CAT TTA CCC GGA GT 3′) were used to amplify the murine IgG2a from genomic DNA whilst introducing a *BstE*II site 5′ of the CH1 and an *Xba*I site 3′ of the stop codon respectively and cloned into the parking vector. pVH-C1-hIgG1 was digested with *Sal*I and blunted with T4 DNA polymerase (New England Biolabs) prior to recovery from gels, and digestion with *Xba*I, into which a *BstE*II blunted/XbaI fragment from the parking vector was ligated to create pVH-C1-mIgG2a.

#### 2.1.4 Construction of pVH-C1-mIgG2b and IgG3

Primer pairs Mg2b-for (5′ ***GAT ATC*** GGT CAC AGT GCA AGC TCT 3′) and Mg2b-rev (5′***TCT AGA*** GCT CAT TTA CCC GGA GA 3′) were used to amplify the murine IgG2b from genomic DNA whilst introducing an *EcoR*V site 5′ of the CH1 and an *Xba*I site 3′ of the stop codon respectively and cloned into the parking vector. pVH-C1-hIgG1 was digested with *BamH*I and blunted with T4 DNA polymerase (New England Biolabs) prior to recovery from gels, and digestion with *Xba*I, into which an *EcoR*V blunted/*Xba*I fragment from the parking vector was ligated to create pVH-C1-mIgG2b. Primer pairs Mg3-for (5′ ***GAT ATC*** GTT CAG GAT AGA GCT GGG 3′) and Mg3-rev (5′ ***TCT AGA*** TCT CAT TTA CCA GGG GA 3′) were used in the same way to generate pVH-C1-mIgG3.

### 2.2 Generation of *Py*MSP1_19_-specific antibodies

#### 2.2.1 Construction of pVL-B10-mouse kappa light chain

pVL-C1-mouse kappa light chain was digested with *ApaL*I/*Xho*I to release the VL of C1. The VL of B10 was subcloned as an *ApaL*I/*Xho*I fragment derived from the parental expression vector pB10-VL/10 (Pleass et al., 2003).

#### 2.2.2 Construction of pVH-B10-mIgG2a

pVH-C1-mIgG2a and parental plasmid pB10-VH/5 (Pleass et al., 2003) were digested with *BssH*II/*BstE*II to release the VH of C1 (pVH-delC1-mIgG2a) and the VH of B10 respectively. The VH of B10 was sub-cloned into pVH-delC1-mIgG2a using the same restriction sites and renamed pVH-B10-mIgG2a.

#### 2.2.3 Construction of pVH-B10-mIgG1

The plasmid pVH-B10-mIgG1 was generated in two steps. pVH-C1-mIgG1 and pVH-B10-mIgG2a were digested with *BssH*II/*BstE*II to release the VH of C1 and VH of B10 respectively. The VH of B10 was sub-cloned into pVH-delC1-mIgG1. Due to the presence of a second *BstE*II site in exon 1 (CH1 domain) 160 bp after the VH of B10 3′ *BstE*II site, both plasmids were digested with *BstE*II and the released fragment sub-cloned into pVH-B10delC1-mIgG1 with the same restriction sites to generate pVH-B10-mIgG1.

#### 2.2.4 Construction of pVH-B10-mIgG2b and IgG3

**Fig. 2:**
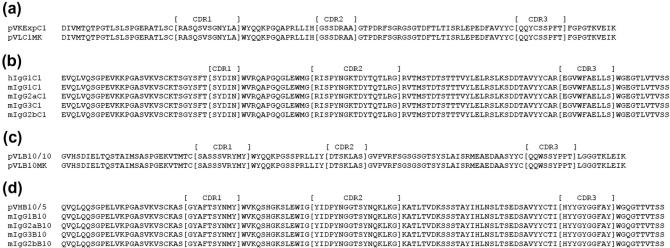
Sequencing of C1 and B10 VH and VL genes. Amino acid sequences alignment of *Pf*MSP1_19_ and *Py*MSP1_19_-binding epitope-matched mouse IgGs with the respective parental expression plasmids. (a) pVL-C1-mouse kappa compared with pVKExpress C1. (b) IgG1, IgG2a, IgG2b, and IgG3-C1 mouse IgG constructs compared with (h)IgG1-C1. (c) pVL-B10-mouse kappa compared with pB10-VL/10. (d) IgG1, IgG2a, IgG2b, and IgG3-B10 mouse IgG constructs compared with pB10-VH/5.

pVH-B10-mIgG2a was partially digested with *Mfe*I/*Xba*I to release the IgG2a (pVH-B10-delmIgG2a) sequence after the VH of B10. IgG2b and IgG3 were released from their respective parking vectors with *EcoR*I/*Xba*I and sub-cloned into pVH-B10-delmIgG2a compatible cohesive restriction site ends (*Mfe*I–*EcoR*I) and *Xba*I. The newly generated expression vectors were renamed as pVH-B10-mIgG2b and pVH-B10-mIgG3. All plasmids were sequenced in both directions to confirm that no errors were introduced into either the variable or constant region genes during PCR/cloning. Amino acid translation of the VH and VL genes for both C1 and B10 expressed in the mouse IgGs confirmed that they are indeed epitope-matched and are shown in Fig. 2.

### 2.3 Protein expression and purification

Chinese hamster ovary (CHO)-K1 or human embryonic kidney (HEK)-293T cells were transfected by electroporation with corresponding heavy- and light-chain plasmids and the Abs were expressed as follows: IgG1 on HEK-293T and IgG2a, IgG2b, and IgG3 on CHO-K1. Positive clones secreting MSP1_19_-specific IgG detected by ELISA, on plates coated with recombinant *Pf*MSP1_19_-GST (Burghaus and Holder, 1994) or *Py*MSP1_19_-GST (Lewis, 1989), or immuno-blotting using goat anti-mouse IgG-Fc (Pierce), goat anti-mouse IgG1, IgG2a, IgG2b or IgG3 (Southern Biotech), or a goat anti-mouse kappa (Southern Biotech) all conjugated to horse radish peroxidase (HRP) as previously described (Pleass et al., 2003). From large-scale cultures, mouse IgG was purified on HiTrap protein G-Sepharose (GE Healthcare) by FPLC. The integrity and purity of the antibodies were verified on 4–12% SDS–PAGE gradient gels (Invitrogen).

### 2.4 Immunofluorescence assay (IFA) and surface plasmon resonance (SPR) analysis

For IFAs, washed erythrocytes from mice infected with the transgenic (Tg) parasite Pb-PfM19 described by de Koning-Ward and colleagues (2003), were fixed on slides in methanol–acetone (1:1, vol/vol) for 10 min. After being blocked in phosphate buffered saline (PBS)/5% (vol/vol) goat serum, slides were incubated with Abs at 5 μg ml^−1^ in blocking buffer for 1 h, washed, then incubated for 1 h with fluorescein isothiocyanate (FITC)-conjugated goat F(ab′)_2_ anti-human IgG (Sigma or Caltag) or goat anti-mouse IgG γ-chain specific (Southern Biotech) diluted 1:500 in blocking buffer. After washing, slides were mounted with DAPI anti-fade and Ab binding examined by IF microscopy on a Zeiss Axioscope 40 microscope. For SPR, quantitative association (*K*_A_) and dissociation (*K*_D_) constants of the mouse IgG subclasses were measured using a BIAcore X Machine (GE Healthcare). Antigen (*Pf*MSP1_19_-GST) at a concentration of 50 μg/ml was amine-coupled onto a dextran matrix CM5 sensor chip using a Pharmacia Biosensor Amine Coupling Kit following the manufacturer’s instructions. Various concentrations of purified IgGs were then injected into a final volume of 50 μL at a rate of 10 μL/min over the chip and binding kinetics analysed using BIAsimulation 3.0 software.

### 2.5 Luminol chemiluminescence assay of respiratory burst and myeloperoxidase release

Neutrophils were isolated from heparinized blood taken from healthy volunteers by the sedimentation of erythrocytes in 6% (w/v) dextran T70 (GE Healthcare) in 0.9% (w/v) saline at 37 °C for 30 min, followed by leucocyte separation on a discontinuous density gradient of Lymphoprep (*ρ* = 1.077 g/cm^3^; Nycomed, Birmingham, UK) over Ficoll-Hypaque (*ρ* = 1.119 g/cm^3^), centrifuged at 700*g* for 20 min at room temperature. Approval for the collection and use of human cells was obtained from the local Queen’s Medical Centre ethics committee. Wells of chemiluminescence microtiter plates (Dynatech Laboratories, Billinghurst, Sussex, UK) were coated with 150 μl of *Pf*MSP1_19_ at 5 μg ml^−1^ in coating buffer (0.1 M carbonate buffer, pH 9.6) and incubated overnight at 4 °C. After washing three times with PBS, 150 μl of anti-*Pf*MSP1_19_ IgG at 100 or 50 μg ml^−1^ was added to antigen coated wells. In each case, triplicate wells were prepared and left for 2 h at room temperature. After washing as before, 100 μl of luminol [67 μg ml^−1^ in Hank’s buffered saline solution (HBSS) containing 20 mM HEPES and 0.1 g/100 ml globulin-free BSA (HBSS/BSA)] was added to each well. After the addition of 50 μl of purified neutrophils (10^6^/ml in HBSS/BSA) to each well, plates were transferred to a Microlumat LB96P luminometer, and the chemiluminescence was measured at 2 min intervals for 120 min at 37 °C. Data were analysed using Excel software.

### 2.6 Passive immunization and parasite challenge

Pathogen-free BALB/c mice from 6 to 8 weeks of age (caged individually) were used to test *in vivo* efficacy of mouse IgG1 in passive transfer experiments. IgG1 was administered (0.5 mg/injection) intraperitoneally (i.p.) on day −1, 0, and +1. Mice were challenged with 5000 parasitized red blood cells (prbc) with Pb-PfM19b that were administered i.p. 5 h after Ab injection on day 0. From day +2 mice were screened daily for weight loss and % infected erythrocytes (parasitemia) counted by blood smears stained with Giemsa (Sigma). At the end of the experiment or when mice lost more than 20% of their initial weight, the animals were humanely sacrificed. All animal experiments were approved by the Home Office and performed in accordance with UK guidelines and regulations (PPL 40/2753).

## 3 Results

### 3.1 Characterization of *Pf*MSP1_19_-specific mouse IgG1, IgG2a, IgG2b and IgG3

**Fig. 3:**
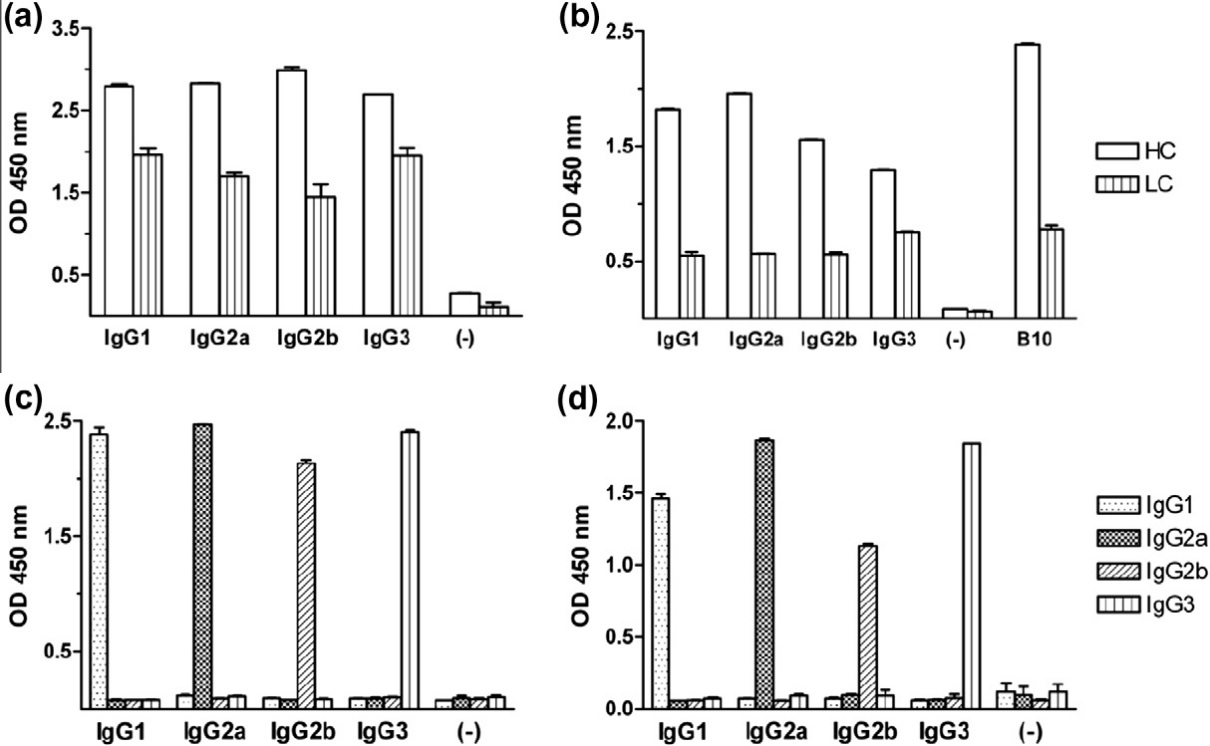
ELISA screening of *Pf*MSP1_19_ and *Py*MSP1_19_-specific mouse IgGs. HEK-293T or CHO-K1 mammalian cell lines were stably-transfected by electroporation with expression plasmids coding for the panel of mouse IgGs targeting *P. falciparum* or *P. yoelii* MSP1_19_. Clones secreting antigen-specific IgGs were detected by enzyme-linked immunosorbent assay (ELISA). Five micrograms of recombinant purified antigen *Pf*MSP1_19_ (a) or *Py*MSP1_19_ (b) was coated and secreting clones detected with an anti-mouse IgG Fc-specific (HC) or anti-mouse kappa light chain (LC) secondary antibodies. To confirm the secretion of the panel of C1 (c) or B10 (d) epitope-matched mouse IgGs a capture ELISA was performed. Plates were coated with goat anti-mouse F(ab′)_2_ and supernatant from secreting clones analysed with subclass-specific secondary antibodies. All secondary reagents used were HRP conjugated and all plates were read at 450 nm. Bars represent the mean OD values from duplicate or triplicate wells. Supernatant from untransfected cells was used as negative control. Two micrograms of mAb B10 was used as positive control on panel b. Error bars represent Standard Deviation (SD).

**Fig. 4:**
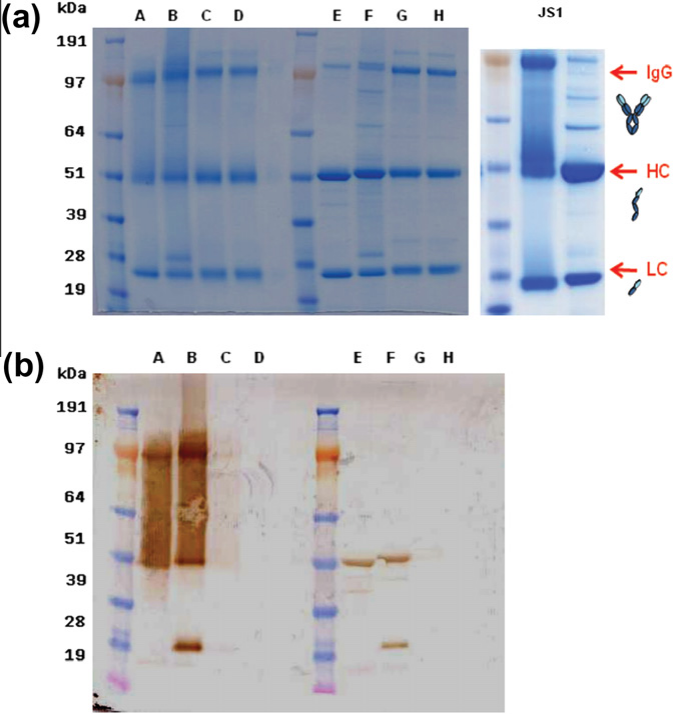
Characterization of purified recombinant *Pf*MSP1_19_-specific epitope-matched mouse IgGs. Characterization of the purified *Pf*MSP1_19_-specific mouse IgGs. KEY: IgG1 (A), IgG2a, (B), IgG2b (C), IgG3 (D). (a) Five micrograms of each Ab was run on NuPAGE 4–12% Bis–Tris SDS gels (Invitrogen) under denaturing non-reducing conditions (A–D) or denaturing reducing conditions (E–H) and compared with the control antibody human IgG1 (JS1) from which they were derived (IDEM). An expected MW of ∼150 kDa for the intact antibodies was shown (arrowed, ‘IgG’) although free constitutive heavy chains and light chain were observed (50 and 25 kDa respectively, arrowed). (b) Western Blot analysis of the IgG samples transferred into PVDF membranes. Analysis was made following the same conditions and sample order described on (a). Murine IgG1, IgG2a-C1 antibodies were clearly detectable with polyclonal anti-mouse IgG Fc-specific secondary antibody compared to IgG2b which was detected very faintly, but none of them with the subclass-specific secondary antibodies used in ELISA.

**Fig. 5:**
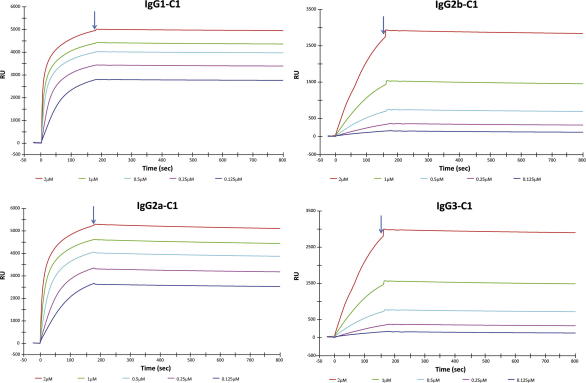
Surface plasmon resonance (SPR)-derived association and dissociation curves of mouse IgGs binding to *Pf*MSP1_19_. SPR association (*K*_A_) and dissociation (*K*_D_) curves of mouse IgG binding to recombinant purified antigen *Pf*MSP1_19_ amine-coupled and immobilized to a CM5 sensor chip. The antibodies were injected into flow at five different concentrations of 2 μM (red), 1 μM (green), 0.5 μM (cyan), 0.25 μM pink), and 0.125 μM (dark blue) at time 0 and replaced with buffer alone at the times indicated with arrows. No binding was seen from any of the IgGs onto control Ag coated in flow cell 2. Data are summarized in Table 1. (For interpretation of the references to colour in this figure legend, the reader is referred to the web version of this article.)

**Fig. 6:**
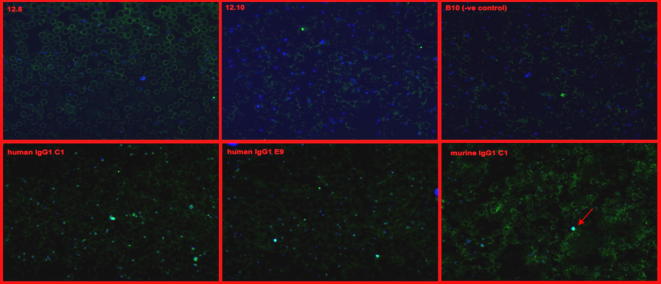
Characterization of purified mouse IgG1-C1 by immunofluorescence assay (IFA). Mouse IgG1 is reactive with MSP1_19_ on methanol–acetone-fixed smears of merozoites and erythrocytes infected with rodent *P. berghei* Tg for *P. falciparum* MSP1_19_ (Pb-PfM19). Top panel: mAbs 12.8 and 12.10 previously described and known not to bind to Pb-PfM19 (Lazarou et al., 2009) and B10 anti-*Py*MSP1_19_ as negative control (Spencer Valero et al., 1998). Bottom panel: Human IgG1-C1 (JS1) and human IgG1-e9 (JS2) as previously described (McIntosh et al., 2007) and murine IgG1-C1 bind Pb-PfM19 infected erythrocytes. Binding was visualized by staining with a FITC-conjugated secondary reagent with nuclei stained with DAPI and assessed by fluorescent microscopy under 40× magnification.

*Pf*MSP1_19_ and *Py*MSP1_19_-specific mouse IgG1, IgG2a, IgG2b and IgG3 were secreted into culture supernatants from stably transfected CHO-K1 or HEK-293T cells. These recombinant antibodies recognized either recombinant *Pf*MSP1_19_ or *Py*MSP1_19_ by ELISA with polyclonal anti-mouse IgG Fc- or kappa light chain-specific secondary reagents (Fig. 3a and b). To check that each cell line only secreted the intended antibody class, a second sandwich ELISA was developed to confirm the subclass of the secreted IgG antibodies (Fig. 3c and d). Here we focus on the *Pf*MSP1_19_-specific mouse IgG1, IgG2a, IgG2b and IgG3 that were purified from CHO-K1 or HEK-293T culture supernatants by protein-G affinity-chromatography and appeared pure by SDS–PAGE analysis (Fig. 4a). Total yields of protein after purification, dialysis, and concentration from numerous different batches of culture supernatant over several time points were in the region of 15 mg from 30 l of supernatant for IgG1, and 2.8 mg, 2.1 mg, 1.5 mg from 7 l of supernatant for IgG2a, IgG2b and IgG3 respectively. The poor yields of IgG2a, IgG2b and IgG3 were most likely a consequence of these subclasses being cloned into a different expression plasmid (pVHExpress) compared to the mouse IgG1 (pcDNA3.1), and therefore the majority of work described focuses on IgG1 for which a sufficient quantity of antibody was derived for *in vivo* studies. The mouse IgG antibodies had an apparent molecular weight (MW) of approximately 150 kDa, although there were minor differences in the MW dependent on subclass. All the preparations contained a significant proportion of free heavy and light chains (arrowed), suggesting that not all the expressed protein folded into intact heterodimeric antibody. Western blotting with the IgG subclass-specific reagents used in the detecting ELISAs were unable to detect any of the mouse IgGs by immunoblotting on both non-reducing and reducing gels, although an alternative polyclonal goat anti-mouse IgG-Fc did recognize mouse IgG1, IgG2a and very faintly IgG2b (Fig. 4b). No significant binding to mouse IgG3 was observed with this reagent despite the presence of an equivalent amount of protein. Importantly, surface plasmon resonance (SPR) analysis for all the IgG subclasses generated revealed no reduction in affinity for *Pf*MSP1_19_ when compared with the parental human IgG1 antibody (Table 1 and Fig. 5), the binding constants remaining essentially the same for each subclass. The recombinant mouse IgG1 by indirect IFA produced a characteristic pattern of MSP1 reactivity from *P. falciparum* infected erythrocytes (not shown) and *Plasmodium berghei* parasites Tg for *Pf*MSP1_19_ (Fig. 6, bottom right corner, arrowed).

### 3.2 IgG1 triggers *Pf*MSP1_19_-specific neutrophil nicotinamide adenine dinucleotide phosphate (NADPH) oxidase activation through FcγR cross-linking

**Fig. 7:**
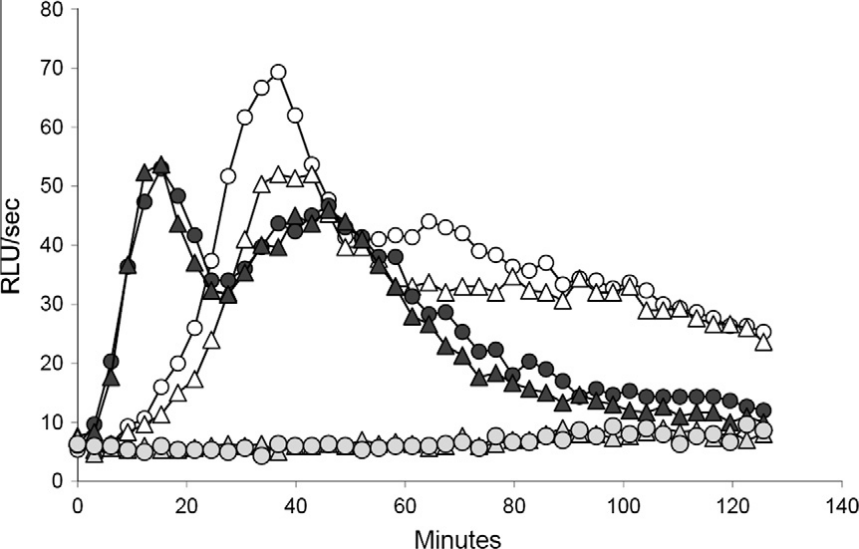
Mouse *Pf*MSP1_19_-specific IgG1 is functional. Neutrophil NADH oxidative bursts were measured by adding antibodies to a chemiluminescence plate coated with recombinant purified antigen *Pf*MSP1_19_. Relative Light Units (RLU, arbitrary units of light produced/second) were calculated from triplicate wells using neutrophils as previously described (McIntosh et al., 2007; Pleass et al., 2007, 2003). Each point in the graph represents the mean value of triplicate wells. RLU were measured for over 120 min after the IgGs were added to neutrophils at two different concentrations of 50 (△) or 100 (○) μg/ml of mIgG1-C1. The overall response was comparable to that derived from human IgG1-C1 at the same concentrations (▴, ● 50 or 100 μg/ml respectively). An IgG2a-Fc fused to antigen (*Pf*MSP1_19_-IgG2a-Fc) was used as an internal negative control (,  50 or 100 μg/ml respectively).

We next assessed the ability of the mouse IgG1 to interact with FcγRs and induce NADPH oxidase activation (respiratory burst) and degranulation in blood neutrophils (Fig. 7). For neutrophils, luminol chemiluminescence provides a read out of NADPH oxidase activation and myeloperoxidase release (Pleass et al., 2007, 2003). When attached to *Pf*MSP1_19_-GST-coated plates, the mouse IgG1 induced comparable respiratory bursts to the parental human IgG1 (Fig. 7), suggesting that binding to *Pf*MSP1_19_ allows mouse IgG1 to be presented to neutrophil FcγRs in an optimal configuration for receptor cross-linking and triggering of functional responses. This finding is similar to that reported previously with a different mouse IgG1 (mAb 12.10) also recognizing *Pf*MSP1_19_ (Lazarou et al., 2009). Since mouse IgG1 is known to bind to human FcγRIIA but not to FcγRIII, the activation of these neutrophils most likely occurs through binding to the former receptor (Pleass et al., 2003).

### 3.3 Passive transfer of mouse IgG1 into wild type mice has no effect on the course of a rodent malaria infection

**Fig. 8:**
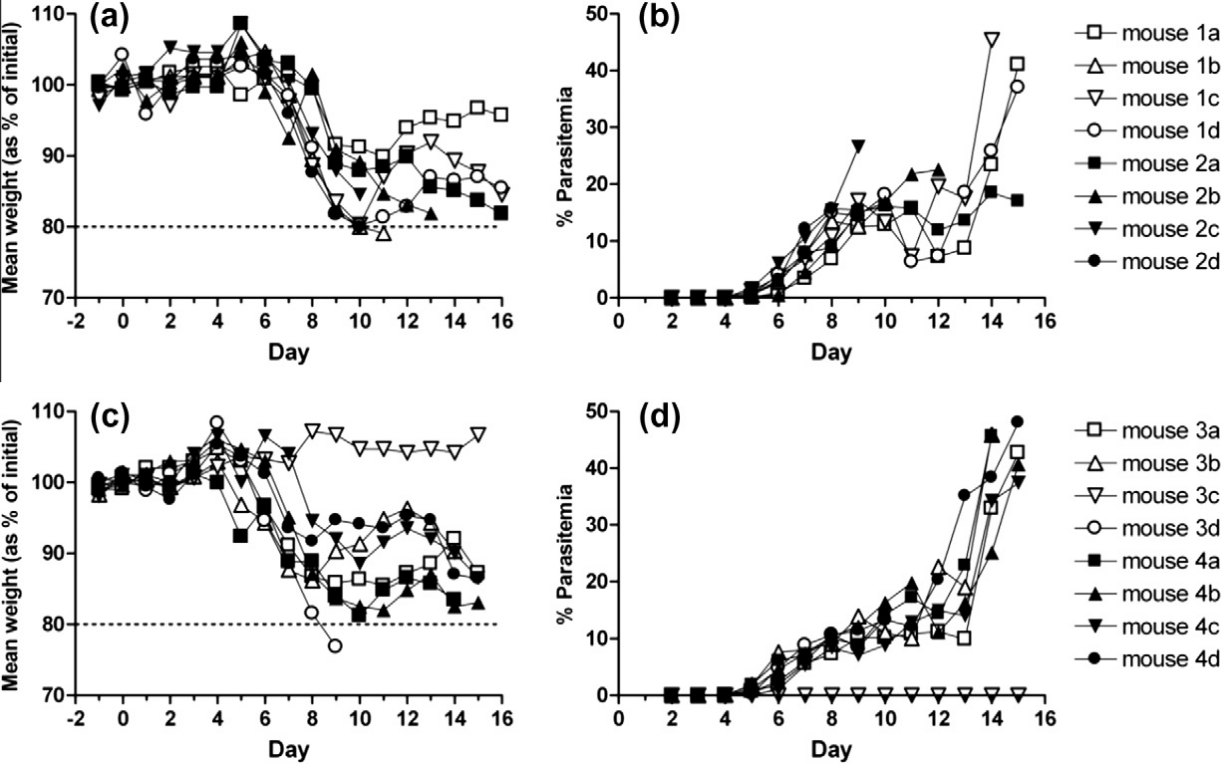
Passive transfer of mouse *Pf*MSP1_19_-specific IgG1 does not protect mice from infection. Groups of four BALB/c wild type littermates were injected i.p. with a total dose of 1.5 mg of purified anti-*P. falciparum* mouse IgG1 (open symbols, groups 1 and 3) or with PBS-vehicle control (closed symbols, groups 2 and 4) and challenged with 5000 parasitized red blood cells (prbc) derived from ‘passage’ animals infected with Pb-PfM19. Similar results were obtained from two independent experiments (a–b and c–d respectively). (a) and (c) Mean weight as % of initial (mean weight from days −1, 0, +1) was assessed daily to determine if an animal had lost more than 20% of its original weight (dotted line) used as a humane end-point in accordance with Home Office regulations. (c) and (d) Seven out of eight animals treated with mIgG1-C1 succumbed to malaria infection and no significant difference was shown between the test and control groups. The percentage of parasitemia between the test and control groups was similar in both experiments.

Despite binding *Pf*MSP1_19_ with high affinity, passive transfer of mouse IgG1 into WT BALB/c mice failed to protect seven out of eight animals, in two separate experiments, from a challenge infection with blood-stage *P. berghei* parasites Tg for *Pf*MSP1_19_ (Fig. 8).

## 4 Discussion

Although we have previously shown that mice Tg for human FcRs are very useful for investigating human antibody function *in vivo*, particularly where there are no suitable animal models for *P. falciparum* infection in humans (McIntosh et al., 2007; Shi et al., 2011), an argument can be made that they are somewhat contrived, and therefore should be compared in parallel experiments in wild type models of murine malaria infection with murine antibodies. Here we describe the construction of two panels of mouse IgG1, IgG2a, IgG2b and IgG3 recognizing identical epitopes in *Pf*MSP1_19_ or *Py*MSP1_19_.

*Plasmodium* spp. merozoites attach to the surface of red blood cells by means of different surface antigens; among these antigens is MSP1 (Holder, 2009). This surface antigen is cleaved twice; the first cleavage takes place at schizogony followed by a second cleavage to reveal *Pf*MSP1_19_ just prior to invasion (Blackman et al., 1994; Morgan et al., 1999). This 19-kDa peptide is relatively conserved with few polymorphic variants and has been described as a major candidate for vaccine development (Pizarro et al., 2003), and continues to be the subject of study for immune therapy (Roussilhon et al., 2007).

These epitope-matched antibodies were generated by cloning variable genes from mAbs previously shown to confer passive protection from rodent malarias (McIntosh et al., 2007; Spencer Valero et al., 1998), into newly generated expression constructs containing mouse IgG1, IgG2a, IgG2b and IgG3 constant region genes respectively. The original plan was to generate the panel of mouse IgGs with the same expression plasmid (pcDNA3.1). However, due to the presence of a *BstE*II site on the C-terminal site of the VH-C1 in the parental human expression plasmid, we could not find a suitable enzyme site with which releases the human IgG1 sequence from the expression vector and subclones all the mouse IgG sequences in. The approach taken to generate the IgG2a, IgG2b, and IgG3-C1 Abs was to sub-clone in the murine sequences in an expression plasmid (pVHExpress) previously used in the expression of anti-malarial Abs (McIntosh et al., 2007). These plasmids were expressed in CHO-K1 or HEK-293T cells, and all eight secreted antibodies generated were shown to bind their respective MSP1_19_ antigen, and were identified as belonging to the correct subclass by ELISA (Fig. 2).

CHO and HEK-293 mammalian cell lines are widely used in the production of recombinant proteins (Wurm, 2004). Predominantly in industry, CHOs have been used to express a wide range of recombinant proteins including antibodies used in the treatment of numerous diseases, including: multiple sclerosis, arthritis and cancer (Hossler et al., 2009; Kelley, 2009). To a lesser extent, HEK-293s have also been used in the industry to produce recombinant antibodies, but alongside CHOs, they have been the subject of study for increased productivity in the field (Wurm, 2004). HEK-293s have been used to express recombinant proteins for studies in neurology, electrophysiology and to produce mAbs amongst other therapeutic proteins (Chen et al., 2011; Thomas and Smart, 2005). Functional IgGs need to be glycosylated in their Fc-fragment in order to have Fc-mediated effector functions (Jefferis, 2005; Walsh and Jefferis, 2006), although we have not directly assessed the glycosylation status of these reagents, CHO and HEK-293 cell lines have been previously shown to effectively secrete Abs with N-linked carbohydrates (Schlenke et al., 2007; Sethuraman and Stadheim, 2006).

The mouse IgG1, IgG2a, IgG2b and IgG3 all bound *Pf*MSP1_19_ with high affinity as determined by surface plasmon resonance analysis (Fig. 4). The panel of IgG antibodies bound with comparable affinity to recombinant *Pf*MSP1_19_ in line with previous observations with other antibodies (McIntosh et al., 2007), and the mouse IgG1 bound to native protein as determined by immunofluorescence microscopy with infected erythrocytes (Fig. 6). Previous results have shown that the parental human IgG1 from which these mouse antibodies were derived is dependent on FcR-mediated recruitment for function, and that inhibition of erythrocyte invasion and/or inhibition of MSP1 processing are/is not the principal mechanism through which this antibody controls parasites (McIntosh et al., 2007).

Typically in passive transfer experiments with rodent malarias, large concentrations of antibody (1.5 mg total dose per mouse, given as three individual doses) have to be given to provide protection. Although we could produce sufficient quantities of all the antibody classes to allow for preliminary *in vitro* comparisons to be made, we could only generate sufficient mIgG1 with which to do *in vivo* work. Intriguingly, passive transfer experiments with this IgG1 antibody failed to protect BALB/c mice from challenge infection with malaria parasites (Fig. 8). Although the mouse IgG1 was fully functional, as determined by its ability to induce NADPH mediated oxidative bursts from human neutrophils, the antibody was ineffective at controlling the development of malaria when passively transferred into BALB/c mice that were challenged with the Tg parasite Pb-PfM19 (de Koning-Ward et al., 2003). In contrast to mouse IgG2a or IgG2b, mouse IgG1 binds preferentially to inhibitory FcγRIIB (CD32B) expressed on murine macrophages, monocytes and B cells (Nimmerjahn and Ravetch, 2006; Pleass and Woof, 2001), but not on neutrophils, offering a possible explanation as to why this subclass was found to be ineffective at controlling the development of a lethal malaria infection when passively transferred into recipient mice.

The similarities of the genomes of the human and rodent *Plasmodium* spp. with their 14 chromosomes, ranging from 23 to 27 Mb, coding for over 5000 proteins from which around 80% of the +5300 genes found in these genomes are orthologous, highlight the importance of murine malarias as models to understand the human disease (Doolan, 2011; Hall et al., 2005; Janse et al., 2010). These genetic similarities have led to the development of targeted gene knock-outs, mutations, fluorescently-tagged, generation of ‘reporter’ or ‘attenuated’ parasites that contribute to the development of reliable vaccines against the disease (Janse et al., 2010). To date, 561 rodent parasite mutants (predominantly *P. berghei*) have been reported (Rodent Malaria genetically modified Parasites DataBase (RMgmDB) http://www.pberghei.eu/ accessed on 11th August 2011).

The use of Tg *P. berghei* rodent parasites expressing vaccine-candidate Ags from orthologous genes of *P. falciparum* has been described for a wide range of proteins and genes, such as the: sporozoite adhesive protein TRAP (Wengelnik et al., 1999), circumsporozoite protein (CSP) (Persson et al., 2002; Tewari et al., 2002), the chloroquine resistance transporter (CRT) (Ecker et al., 2011), and the UIS4 and HT1 genes that code for a parasitophorous vacuole membrane protein in sporozoites and liver stages (expressed in a Tg *P. yoelii*) and a hexose transporter, respectively (Blume et al., 2011; Mackellar et al., 2010).

Parasites Tg for MSP1_19_ are no exception. Rodent parasite *Plasmodium chabaudi* MSP1_19_ has been expressed in *P. falciparum* and used for *in vitro* studies (Corran et al., 2004), and two Tg rodent *P. berghei* parasites expressing *Pf*MSP1_19_ have been described: Pb-PfM19 and PfMSP1–19Pb_8.7_ (Cao et al., 2009; de Koning-Ward et al., 2003) (respectively). Passive immunization of human IgG1 anti-*Pf*MSP1_19_ in BALB/c mice challenged and infected with Pb-PfM19, protected CD64-Tg mice (but not WT mice) from the course of infection (McIntosh et al., 2007). Similar results were found in mice immunized with purified rabbit anti-*Pf*MSP1_42_ IgG where sterile protection was also observed (Sachdeva et al., 2006). However, alternatively from these experiments, passive transfer of Abs 12.8 and 12.10 into BALB/c mice challenged with Pb-PfM19 confirmed their inability to protect *in vivo* suggesting that the presence of six amino acids of *P. berghei*’s MSP1_19_ in the Tg parasite was required for both mAbs to be able to bind to the *Pf*MSP1_19_ epitope (Lazarou et al., 2009). These discrepant results from passive transfer experiments in infections with Pb-PfM19 have encouraged us to assess the efficacy of the alternate panel of IgGs targeting *Py*MSP1_19_ to elucidate their response in a more natural model of infection.

From 1965 to 2010, more than 1900 immunization studies have been conducted involving all four rodent *Plasmodium* spp. and human parasites in feasible vaccine development trials. In this vast amount of time and research, many combinations of treatments have been studied such as: (1) immunizations with live, attenuated or dead parasites, (2) purified proteins such as Ags, Abs, DNA subunits, via multiple routes of delivery (Guilbride et al., 2010). Passive transfer experiments *in vivo* looking at the efficacy of Abs in controlling malaria continue to be of interest as still, the mechanisms of action of the Fc-portion of Abs in controlling the disease is not completely understood. Current improvements in the mass production of recombinant proteins could allow IgG therapies to become the standard choice of treatment against blood-stage malaria in the near future. It is of our interest to continue this project and aim to increase the yield of purified Abs from transfected mammalian cell lines by expressing our panel of IgGs in Sp2/0 myelomas for instance. With greater yields of purified Abs, we would be able to complete the *in vitro* characterization of the remaining -C1 Abs and compare *in vivo* both panels of the epitope-matched IgGs constructed.

In summary, two panels of epitope-matched mouse IgGs (IgG1, IgG2a, IgG2b, and IgG3) targeting *Pf*MSP1_19_ or *Py*MSP1_19_ were constructed and expressed in mammalian cell lines. A novel *Pf*MSP1_19_-specific mouse IgG1 did not show protective capability against parasite challenge in mice but was shown to be fully functional *in vitro*. Whilst this finding may indicate that IgG1 does not play a major role in protection against malaria, we cannot rule out the possibility that the findings reflect certain shortcomings of the *Pf*MSP1_19_ Tg *P. berghei* rodent malaria model used, hence future work aims to investigate the role of the other IgG subclasses generated using the anti-*P. yoelii* panel of antibodies described herein.

## Figures and Tables

**Table 1:** Association (*K*_A_) and dissociation (*K*_D_) rate constants for the mouse (m) and human (h) IgG antibodies measured by SPR analysis.^a^

Antibody	*K*_A_ (1/M)	*K*_D_ (M)
mIgG1-C1	4.48 × 10^7^	2.3 × 10^−8^
mIgG2a-C1	3.32 × 10^7^	3.01 × 10^−8^
mIgG2b-C1	1.2 × 10^7^	8.41 × 10^−8^
mIgG3-C1	2.33 × 10^7^	3.11 × 10^−8^
hIgG1-C1	3.94 × 10^7^	2.3 × 10^−8^

a Methodology described in McIntosh et al. (2007) and Pleass et al. (2003).
